# Factors influencing recycled materials using in the construction industry in China: an application of the extended theory of planned behavior

**DOI:** 10.3389/fpsyg.2025.1669511

**Published:** 2026-02-05

**Authors:** Yidan Zhu, Chenxi Zhao

**Affiliations:** 1 School of Housing, Building and Planning, Universiti Sains Malaysia, Penang, Malaysia; 2 School of Mathematical Sciences, Applied Science (Mathmatics), Universiti Sains Malaysia, Penang, Malaysia

**Keywords:** use of recycled material, intention to recycle, recycling behavior, Theory of Planned Behavior, construction industry of China

## Abstract

Waste from the construction sector is a significant component of the global waste composition of many continents. There is a lack of research on the attitude and behavior of workers toward recycling. Although changing workers’ attitudes and behaviors toward recycling in attaining sustainable waste management in the construction industry is a tall order, it is crucial to identify. So, the aim of this study is to comprehensively understand workers’ psychology by emphasizing new factors that can measure their intention to recycle and recycling behavior using the extended Theory of Planned Behavior. This study selects workers of the construction industry of China to assess their recycling behavior by testing the relationships among the proposed study model variables. Past research based on the Theory of Planned Behavior (TPB) seldom offers new determinants, such as consequences/Outcomes of Recycling (COR), or investigates demographic factors (age, experience, and gender). This leaves a gap in comprehending the psychological and contextual frames behind recycling in the construction industry. A total of 359 respondents, including Chinese workers, were surveyed and returned the data for further analysis using SPSS and AMOS. The statistical techniques employed are the confirmatory factor analysis. The findings of this study revealed that attitude, perceived behavioral control, and consequences of recycling significantly influenced intention to recycle and recycling behavior among the construction workers. Notably, subjective norms and past recycling behavior were not significant predictors. Moreover, age and experience also significantly influenced the recycling behavior, while gender did not. The study’s main contribution lies in extending the TPB framework within the context of the Chinese construction industry, introducing and validating new determinants (COR, CS) that provide a deeper psychological understanding of recycling behavior. Practically, the results prioritize critical elements for stakeholders, suggesting that managerial and policy interventions should focus on cultivating positive attitudes, enhancing perceived control through infrastructure and support, and leveraging outcome awareness, particularly among younger and less experienced workers, to bridge the implementation gap in construction waste recycling.

## Introduction

The need to deal with climate change around the world has made sustainable development a top priority for national strategy. China has promised to reach a carbon peak by 2030 and carbon neutrality by 2060 (Wang et al., 2025b; Wang et al., 2024). This mandate places intense scrutiny on resource-intensive sectors, particularly the construction industry, which is characterized by high investment, high energy consumption (40% of national energy), and high pollution (about 38% of greenhouse gases and 30% of construction waste (Wang et al., 2025a). The global construction industry has increasingly adopted sustainable practices, such as recycling waste materials to reduce the consumption of natural resources and monitoring the embodied carbon in raw materials (Ajayi et al., 2015). These initiatives not only reflect institutional and environmental priorities but also influence the attitudes of construction workers toward sustainable practices. The world is getting more urbanized, with almost 50% of its people residing in cities. Even though cities contribute 80% of the world’s GDP, they consume 75% of its resources and emit 80% of its Carbon Dioxide (Kapoor and Gupta, 2024). Hence, urban sustainability is essential (Ma and Zhang, 2020). To ensure quality of life for the urban dwellers. With each passing year, the amount of environmental trash increases alarmingly because of the expanding worldwide population and the increased consumption of manufactured goods (Rhodes et al., 2014). The ever-increasing amounts of garbage, therefore, cause concerns to local governments, especially in terms of efficient garbage disposal and their waste management system. The United Nations’ Sustainable Development Goals (SDGs) overriding strategy for urbanization is sustainable development, whereby the environment in cities should be protected against pollution Kaza et al. (2018). It is expected that by 2050, the world will generate nearly 3.40 billion tons of waste every year. Record released by Bai and Lin (2022). Shows that in 2019, China alone generated 242 million tons of garbage. Similar worrisome statistics and per capita data have also been recorded in the majority of other industrialized countries. In terms of the generation of construction waste globally, “What a Waste 2.0” published by the World Bank, reported that in 2018, China was the notorious first offender, followed by the United States. China outperformed the United States by almost three times, more than the combined GDP of another six countries. In fact, the “garbage siege” problem had affected almost two-thirds of the Chinese towns in China itself (Wang et al., 2022).

**FIGURE 1:**
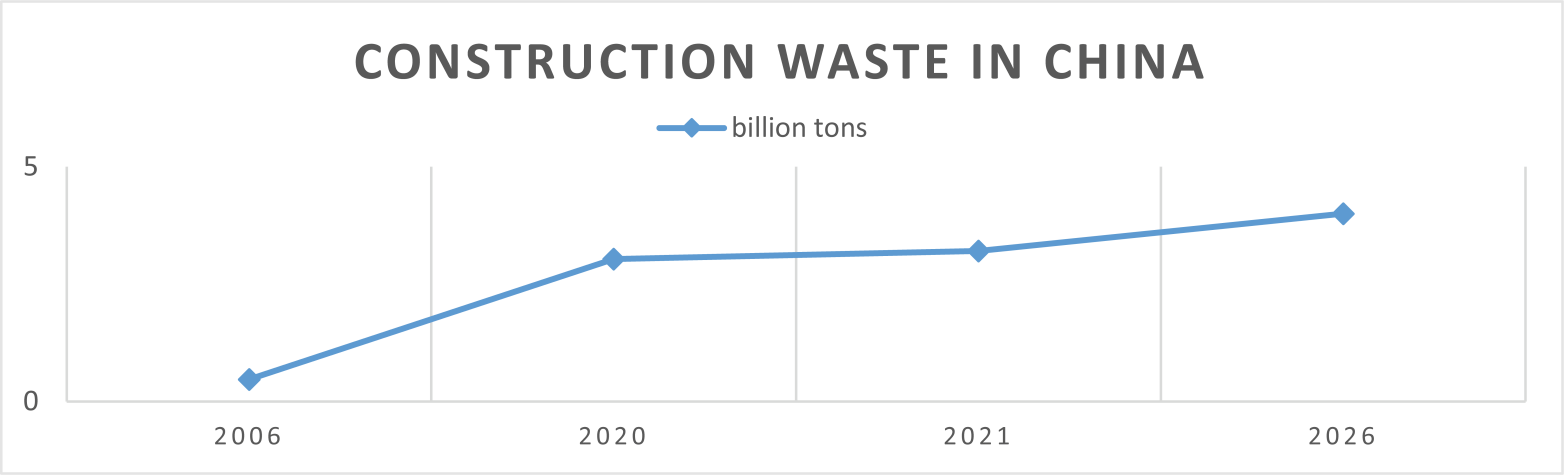
Construction waste has been rapidly increasing in the past decade and is forecasted to rise to 4 billion tons by the year 2026. China Construction and Demolition Waste Disposal Industry Market Report 2021.

Construction waste generation is increasing rapidly along with urbanization and industrialization, and the negative environmental issues associated with it are endangering towns and villages around the world from expanding sustainably (Mak et al., 2019; Pakpour et al., 2014). Generally, the generation of construction waste in the construction industry includes waste generated during design, construction, demolition, and the entire construction life cycle. In terms of stages of construction, residential buildings, non-residential buildings, and underground buildings produce wastes from various materials such as concrete and drywall waste streams. In fact, the wastes produced from each of these sources actually relate to the type (Wang et al., 2022) and the method of the building projects (Li and Zhang, 2013) and according to Bakchan and Faust (2019) Will finally influence the type of construction waste management and automatic quantification system needed. Researchers employ models such as the three-stage SBM-DEA model to assess the efficiency of construction waste and carbon reduction (CWCR) at the regional level, treating construction waste (CW) and carbon emissions (CE) as undesirable outputs (Wang et al., 2024; Wang et al., 2025b). An analysis from 2010 to 2020 shows that overall CWCR efficiency is getting better, but there are still big differences between regions (Wang et al., 2024). Unfortunately, the problem of construction waste management has emerged as a major barrier to the advancement of a “Zero Waste World” and ecological civilization, especially in China (Wang et al., 2022). Figure 1 shows how the construction waste has been rapidly increasing in the past decade, and it is forecasted to rise to 4 billion tons by the year 2026. Therefore, it is necessary to implement targeted policies for effective construction waste management, especially in China, urgently. This can be done through aggressive recycling measures, as construction trash definitely pollutes the environment, and construction activities consume resources indiscriminately.

It is generally agreed that the “3R” (Reduce, Reuse, and Recycle) approach should serve as a guide for the Construction and Demolition Waste (CDW) management procedures (Huang et al., 2018). The effectiveness of adopting such measures is currently relatively ineffective in China (Yuan et al., 2022). Despite the fact that construction waste makes up between 30 and 40% of all municipal garbage. Unfortunately, it has been found that less than 5% of the construction waste is recycled or reused in China (Huang et al., 2018) Comparatively, several industrialized nations such as the US, Japan, South Korea, Denmark, and Germany are active in recycling, as evidenced by their reuse rates of construction wastes that range from 70 to 95%. Although experts in China have been paying close attention to addressing the issue of the poor recycling and low reuse rates since the early 1990s, waste management in the construction industry has not improved much (Hua et al., 2022; Jin et al., 2017).

Present research is grounded in the Theory of Planned Behavior (TPB) (Ajzen, 1991), an extensive framework for the prediction of behavior intentions from the attitudes, subjective norms, and perceived behavioral control that shape as precipitating factors. TPB is applied in various domains such as consumer decision-making, environmental practices, and technology adoption (Lee et al., 2010; Tommasetti et al., 2018; Mohamad et al., 2022). In the context of recycling, TPB provides a structured framework to identify how core components influence the willingness to engage in sustainable behaviors.

Although it is useful, TPB has some limitations. For instance, the decision to recycle is typically influenced by the context, which goes beyond the will of a single person, i.e., the availability of the necessary infrastructure, financial incentives, or social obligations, and the Theory of Planned Behavior (TPB) in its standard form may not be able to capture these factors fully (Strydom, 2018; Mohamad et al., 2022). Consequently, researchers have expanded the model by adding new constructs to it for greater explanatory strength (Ajzen, 1991; Tommasetti et al., 2018).

The present research has been developed following the same pattern by extending TPB with the items Consequences /Outcomes of Recycling (COR) and Consequences to Sustainability (CS). In fact, whereas TPB depicts intention as the immediate driver of behavior, the identification of COR and CS allows that, in the long run, ideas about environmental consequences and resource outcomes can significantly influence recycling behavior. So, this union offers a deeper insight into the recycling behavior of those people who live in complicated organizational and industrial milieus.

This expanded framework is a perfect fit for the Chinese construction industry, and it is really a “now is the time” application. The waste from construction and demolition has become a severe environmental problem. However, the recycling rate of such waste in China remains lower than 5%, whereas that of several developed countries ranges from 70 to 95% (Hua et al., 2022; Jin et al., 2017). The authors of this paper investigated not only the Conclude TPB constructs but also the new COR and CS concept, with the aim of significantly closing the implementation gap in the recycling of construction waste and to provide theoretical, as well as practical contributions toward the management of sustainable waste in practice. Subsequently, this study also utilizes the Extended Theory of Planned Behavior (TPB).

In line with this, the main aim of the current study is to determine the factors impacting the use of Recycled Materials in the Construction Industry of China. To achieve the study’s aim, the following Research Objectives are considered. They are:

- To find out the determinants or factors that affect the use of Recycled Materials behavior in the Construction Industry of China.
- To investigate the impact of Attitude (AT), Subjective Norms (SN), and Perceived Behavioral Control (PBC) on the intention to use recycled construction materials.
- To examine the impact of outcomes of Recycling and Past Recycling Behavior (PRB) on the Intention to Recycle (IR) of construction materials.
- To investigate the impact of IR on Recycling Behavior (RB).
- To study the impact of recycling usage of materials and determine the Consequences/ Outcomes of Recycling (COR) to sustainability.
- To investigate the predictive role of socio-demographic factors in recycling behavior.

## Literature review

The TPB framework asserts that human actions are mostly predicted by their intentions. As mentioned by Ahmed and Rashid (2025), perceived behavioral control had a greater influence on zero-waste behavior than environmental attitudes. These findings confirm that normative views and control beliefs, as conceptualized in TPB, are vital psychological mechanisms for understanding and promoting pro-environmental behaviors in waste management practices. According to the theory, the perception of the behaviors and expectations of significant referent people, along with the desire to conform to those referents, constitutes the second type of influence. The elements that are collectively engaged with other easily recalled normative ideas provide a felt social pressure or Subjective Norm toward partaking in the activity, also known as normative views. The third category of influence is controlled beliefs. This focuses on the notion that certain factors may have an impact on a person’s ability to carry out the activity (Ajzen, 2015). According to Bandura (1977), behavioral control arises due to controlled beliefs along with the perception of these components’ ability to support or interfere with behavioral performance (Wang et al., 2024).

Studies applying TPB in this context, suggest that intentions to protect environment such as beliefs in resource conservation, reduced landfill use, and recycling, enhance the intention to adopt recycle and waste management It is assumed that intentions will translate into actions in as much as individuals are actually capable of doing so, that is, in so far as they have actual control over the behavior. Thus, it shows that behavioral control will temper how intention affects behavior (Ajzen, 2015). Ajzen (1991) later postulates that there are definitely three primary factors that these intentions are based on. They are Attitudes (AT), Subjective Norms (SN), and Perceived Behavioral Control (PBC). Positivity or negativity toward the conduct, the perception of others’ expectations, and perceived behavioral control are all aspects of attitude toward behavior (i.e., perceived as one’s own capability to successfully exhibit the behavior). Hence, when there are a favorable AT and SN toward engaging in the targeted behavior, as well as when there is a greater sense of control, a person is more likely to form an intention to do so.

Extending the TPB framework by adding other elements has higher explanatory power. Overall, the components of the internal drivers entailed in the extended TPB framework comprise of eight constructs namely Attitude (AT), Subjective Norm (SN), Perceived Behavioral Control (PBC), Past Recycling Behavior (PRB), Intention to Recycle (IR), Consequences to Sustainability (CS), Consequences/Outcomes of Recycling (COR) and Recycling Behavior (RB). These constructs could mitigate environmental disasters resulting from the ever-increasing Construction and demolition waste recycling behavior scenarios in China.

### Attitude

The TPB refers to one’s perspective or evaluation of engaging in the target activity (Ajzen, 1991). Individuals form their attitudes based on their perceptions of the behavior and the intended recipient of the behavior. Similarly, when an individual performs Recycling Behavior (RB), he connects an object with specific traits (such as with other objects, features, or situations) in order to develop a belief about the object (Ajzen, 2015). The degree to which a person views the behavior in question, whether favorably or negatively, is therefore referred to as their AT toward that behavior. An AT is a feeling-based idea that makes a group of actions more susceptible to a particular set of social circumstances (Liu et al., 2020). Hence, behavioral beliefs or AT are convictions about potential outcomes or other behavioral characteristics. In the context of RB, the beliefs can be related to the technical characteristics of recycling and also to the perceived environmental impact associated with recycling as they may perceive its relative advantages (Sun et al., 2018). Scholars are of the view that knowledge and risk-taking also have a connection to AT formation (Bran and Vaidis, 2020). As opposed to people who are less inclined or less willing to take a chance, risk-takers are predicted to be more likely to promote a new piece of content or engage in novel behaviors (Superti et al., 2021).

AT strongly influences environmental behavior in a favorable way. This is evident from the prior studies. For instance, Ma and Zhang (2020). Found that A has a significant impact on one’s propensity to engage in RB of construction wastes. Wan et al. (2021) conclude that AT is the determining recycling intention. According to them, spreading recycling education through community campaigns or programs can alter people’s AT. Meanwhile, Tonglet et al. (2004b). Accentuate that receiving adequate incentives, having easy access to disposal facilities, being knowledgeable about recycling, and the absence of any physical barriers all have an impact on people’s attitudes about Recycling. Greaves et al. (2013) state that adopting a Recycling mindset can be influenced by a positive view of recycling, such as the importance of proper garbage disposal as an ethical effort to carry out. Thus, all these arguments by previous researchers imply that TPB maintains that knowledge influences attitude by leading to the formation of well-informed opinions regarding beliefs on Recycling and Sustainable waste management systems. In the specialized, labor-intensive ship recycling industry, sustainable development is significantly influenced by human factors (Zhou et al., 2024). Studies show that workers’ environmentally friendly and safe behaviors are directly or indirectly affected by their attitudes, subjective norms, safety awareness, perceived behavioral control, and perceived susceptibility. This information is useful for making environmental policies in that area (Zhou et al., 2024).

### Subjective norms

Subjective Norms or Culture is defined as the “means of categorizing the experience” and relates to the “human group’s particular manner of interpreting the human-made element of the environment.” It is comprised of social norms, roles, and values (Superti et al., 2021). According to another definition, SN forms self-instruction to do what is judged to be right and considered suitable by the members of a community in specific situations (Ajzen, 2015). They are evaluated by having people rate the likelihood of approval or disapproval for engaging in a specific behavior in the form of prescriptive social influence. The term “Subjective Norm (SN)” refers to behaviors, expectations, and other aspects that may be advantageous or disadvantageous in a social group (Mohamad et al., 2022). SN is a major predictor of RB due to its higher possibility of including components of moral and social duty (Kochan et al., 2016). In order to establish SN in the construction recycling environment and affect the behavior, Wang et al. (2018). Stress that proper e-waste recycling policies and regulations should be implemented. Therefore, the SN may allude to what the people believe one should do. In the context of Recycling, factors such as whether the concept is important to the people or not, and whether they believe that they should either use or suggest reusing recycled materials in their construction projects are relevant. These norms could be their preference for environmental issues that can motivate the individuals to develop their Intention to Recycle (IR) and also engage in Recycling Behavior (RB) (Strydom, 2018). According to Tonglet et al. (2004a), an individual’s experiences with SN and sense of social responsibility can have an impact on the growth of RB. Meanwhile, according to several earlier studies, SN essentially has a positive impact on RB in the waste management of the construction sector (Liu et al., 2020; Mak et al., 2019; Wang et al., 2022).

### Perceived behavioral control

Perceived Behavioral Control (PBC) refers to the perception of one’s own skills based on prior knowledge and anticipated barriers to take the desired action. It refers to how simple or difficult a person thinks a particular behavior will be to accomplish certain things. As a result, PBC can alter depending on the circumstances and the action (Wang et al., 2018). Self-efficacy describes how readily or with difficulty one can engage in the behavior, while perceived controllability describes the perception that one has control over the action. These two factors are said to make up PBC (Echegaray and Hansstein, 2017). Since, in addition to predicting behavioral intention, PBC also predicts behavior, Mohamad et al. (2022). Portray that PBC is a key sign of RB, as it depicts the confidence with which a person carries out a specific action. According to prior research, people who are confident in their ability to perform recycling activities are more likely to engage in RB than people who feel that they have little control over current or foreseeable barriers (Tonglet et al., 2004a).

On the other hand, Echegaray and Hansstein (2017) believe that the presence of nearby disposal facilities may affect PBC in recycling, i.e., by giving consumers the notion that they may save time while engaging in e-waste recycling operations. However, another study (Wang et al., 2022) indicates that the primary factor utilized to assess PBC is the experience of recycling compared to those who have never engaged in it. In this study, the sample respondents were asked to rate how easy or difficult they perceived it to be for them to engage and promote RB and to measure PBC. PBC (Perceived Behavioral Control) among TPB constructs is a key and most reliable single factor that has been identified to predict actual recycling behavior. It is a concept that covers both perceived self-efficacy and the degree of controllability over the execution of recycling actions (Ajzen, 2015). In a decomposed TPB model applied to C&D (Construction & Demolition) waste, PBC was a major influence on intentions and behavior, particularly when it was combined with the enabling infrastructure, such as waste sorting bins and regulatory support (Mak et al., 2019). Similarly, Liu et al. (2020) have also pointed out that PBC is the main factor in urban households that drives recycling behavior, emphasizing that practicality and the availability of facilities are the major factors that influence the decision to recycle.

### Intention to recycle

Intentions for engaging in a particular behavior are a sign of “how hard people are willing to attempt to engage in a particular behavior” (Ajzen, 1991). In other words, if the individual may decide at will to execute or not to perform the behavior, intentions may result in the implementation of that behavior. Therefore, Intention to Recycle (IR) comes from PBC, which also influences the behavior itself (Nigbur et al., 2010; Superti et al., 2021). Additionally, research findings recognize that in some circumstances, intentions to engage in a behavior may be impacted by socially acceptable reactions or propensities to provide explanations that are consistent with societal expectations (Mak et al., 2019; Tommasetti et al., 2018). Recent research found that for both the household (Ioannou et al., 2013; Strydom, 2018) and construction recycling (Superti et al., 2021; Wan et al., 2021; Wu et al., 2017) Positive correlations between behavioral intention and actual behavior exist. This shows that under the paradigm of TPB, the desire to promote recycling results motivates the individuals to engage in such Recycling Behavior whenever an opportunity is present for it. this study, IR is measured through assessing whether employees plan to recycle in the coming 3 months or not.

### Consequences/outcomes of recycling

The outcome or impact of a behavior is referred to as the consequence (Triandis, 1979). Consequences set off a feedback cycle that influences both the person who carried out the action and outside parties (conceptualized as the social network) (Superti et al., 2021). The first step of a person’s psychological reaction to a change in a place is awareness of the consequences (Wan et al., 2021; Zhang et al. (2021). Suggest that the minds of individuals may be stimulated for RB by presenting the benefits in a convincing way. These recent studies support what was previously portrayed by Lindsay and Strathman (1997) and Thøgersen (1996) In that people weigh the pros and cons of a certain behavior and analyze the perceived likelihood of negative consequences before making the final decision. This shows that a person’s direct experience with the change in a place may serve as the catalyst for this awareness of the positive and negative consequences. In the current study, the impact of the COR behavior is evaluated by asking the employees about their “propensity to recommend recycling behavior” and “Feedback on the recommendation of RC” from the stakeholders.

### Consequences to sustainability

Sustainable development (SD) emphasizes the integration of three sustainability pillars, namely social, economic, and environmental activities (Ghafourian et al., 2021). Sustainable building results from the integration of SD into the construction industry. When viewed from the balance between the economic, environmental, and social point of view, the process involves making a sustainable and healthy environment where structures and infrastructures have a positive influence on the ecological systems (Starik and Kanashiro, 2013). Since sustainability is a hot topic of many studies owing to it significant impact on the environment, its consequences are important to understand (Mak et al., 2019). Other studies by Bandura (1977); Knussen and Yule (2008); White and Hyde (2012) suggest ways to promote recycling materials for sustainable consumption. Nguyen et al. (2017) are of the opinion that people may be encouraged to recycle wastage by creating awareness so that the negative consequences to environment may be avoided. In another study by Janmaimool (2017), it is said that scholars used the Protection Motivating Theory to encourage individuals regarding how the recycling and waste management behaviors can have positive consequences on the surroundings. Therefore, recycling the construction materials have been promoted as a solution able to deliver social, environmental, economic, and cultural benefits. It has also become one of the increasingly common ways adopted by businesses aiming to conquer/maintain significant market shares. In this study, CS is measured by how respondents feel recycling will have positive effects on the environment.

### Past recycling behavior

Few researches have looked at past behavior in relation to household waste behaviors, yet it may be a significant predictor of recycling intention and behavior (Pakpour et al., 2014; Sommer, 2011). A study by Pakpour et al. (2014) discovered that household waste behaviors in Iran were strongly predicted by moral duty, self-identity and historical behavior. In an earlier research, past behavior was found to significantly correlate with the behavior, thus supporting the TPB (Nigbur et al., 2010). Other studies by Biswas et al. (2000); Knussen and Yule (2008); White and Hyde (2012) maintain that Past Recycling Behavior (PRB) and self-perceptions are predicting factors for the RB among consumers. Meta-analysis conducted by Geiger et al. (2019) confirm that PRB is strongly related to RB, while Li et al. (2019) discovered that previous recycling practices are carried forward into the future and individuals have more intention and engagement in pro-environmental behaviors, including recycling. Hence, PRB is considered as an important determinant of RB in the extended TPB and in this study they are measured by asking the respondents on how often they engaged in RB in the past 3 months.

### Socio-demographic attributes (age, gender and experience)

For this study, the socio-demographic attributes of the construction employees (the sample respondents) include age, gender and years of experience. Over the years, contradictory results have been discovered as far as socio-demographic attributes are concerned. For example, Weigel (1977) attributed recycling to, demographic variables but another study showed no indication of a direct association between demographic factors and behavior (Strydom, 2018). For this study, age, gender and experience are also examined to see whether socio-demographic attributes play a direct role in RB.

Several studies have validated the TPB framework in the context of recycling behavior in both household and organizational settings (Mohamad et al., 2022; Strydom, 2018). On the other hand (Hua et al., 2022; Huang et al., 2018; Wang et al., 2019) did research about waste management in China but they don’t use TPB or SEM in their study. However, many of these studies focus predominantly on household waste management or consumer recycling, with limited attention given to the construction sector, particularly in the context of emerging economies like China. This presents a significant research gap that this study seeks to address. This study directly works on such factors as knowledge of the environment, personal and social norms or institutional/regulatory effects, as opposed to the direct application of the Theory of Planned Behavior (TPB) using Structural Equation Modeling (SEM). Although these studies are informative regarding the issue, they fail to directly look at the mediating effects of factors related to recycling behavior or sustainability results. In order to fill this gap, our research framework builds on the existing approaches by incorporating two new constructs, Consequences/Outcomes of Recycling (COR) and Consequences to Sustainability (CS). This allows us not only to capture immediate behavioral intentions but also the more global sustainability implications to provide a more holistic picture of recycling behavior.

### Hypotheses

**FIGURE 2:**
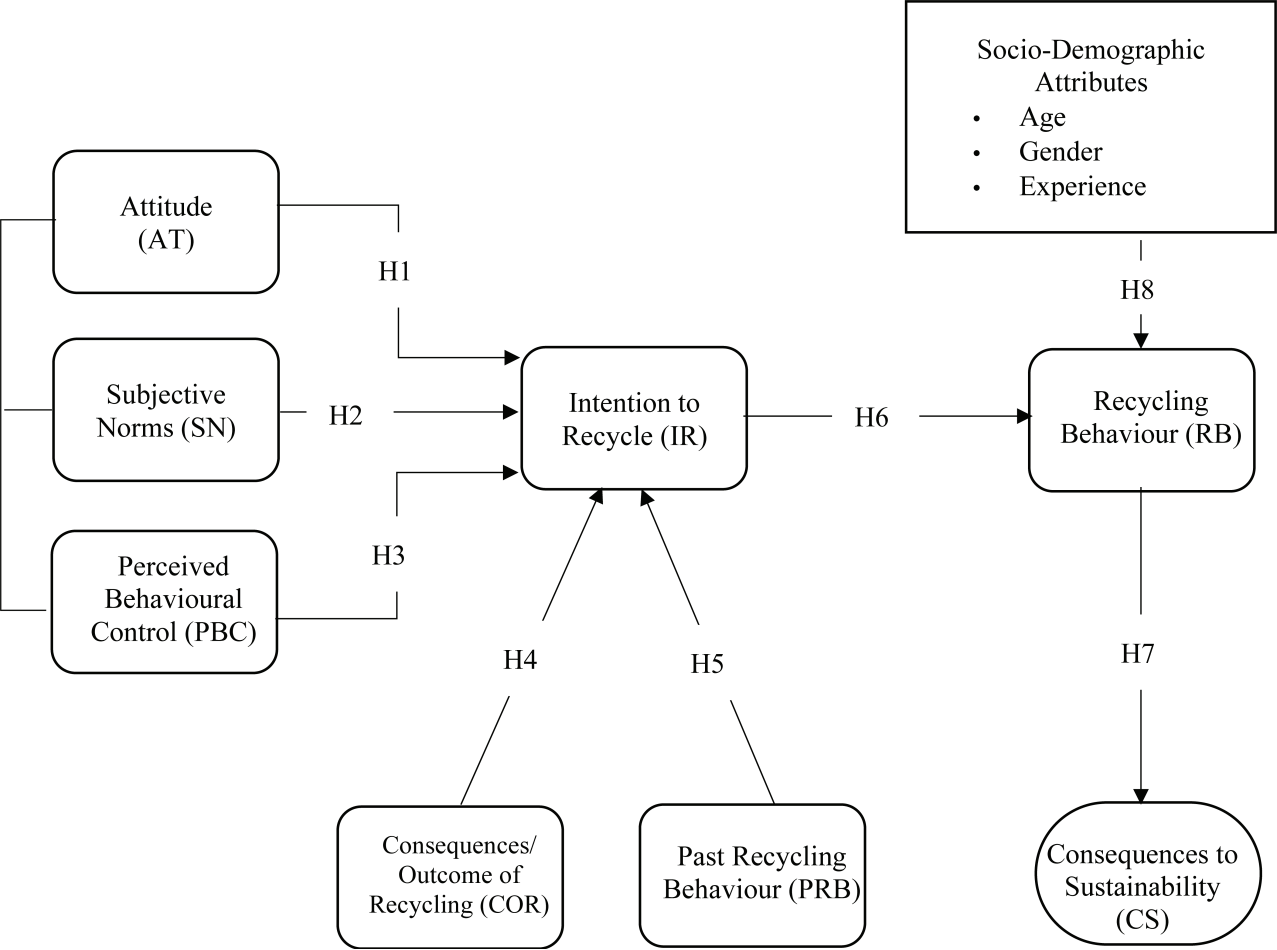
Research model.

Based on the above discussions and employing the extended conceptual framework of the TPB constructs (as shown in Figure 2), eight hypotheses are deduced as follows:

*H1*: Attitude (AT) significantly and positively influences Intention to Recycle (IR).

*H2*: Subjective Norms (SN) significantly and positively influences Intention to Recycle (IR).

*H3*: Perceived Behavioral Control (PBC) significantly and positively influences Intention to Recycle (IR).

*H4*: Consequences/Outcome of Recycling (COR) significantly and positively influences Intention to Recycle (IR).

*H5:* Past Recycling Behavior (PRB) significantly and positively influences Intention to Recycle (IR).

*H6*: Intention to Recycle (IR) significantly and positively influences Recycling Behavior (RB).

*H7*: Recycling Behavior (RB) significantly and positively influences Consequences to Sustainability (CS).

*H8*: Demographics (age, gender and experience) significantly influence recycling behavior (RB).

## Research methods

### Research design

This study employed a descriptive and cross-sectional research design using a quantitative approach. The cross-sectional design was selected to capture the perceptions and behaviors of construction workers at a specific point in time regarding recycling practices. This design is appropriate for exploring the relationships between psychological constructs within the extended Theory of Planned Behavior (TPB) framework. The research approach is deductive, aiming to empirically test the proposed hypotheses based on established theory

### Population and sampling

The study was executed on the target population, which consisted of employees working in the construction industry across six regions in China: Zhengzhou, Kaifeng, Luoyang, Hangzhou, Suzhou, Shanghai, Nanjing, and Xian. These regions were selected due to their high construction activity and varying levels of urban development, providing a diversified view of recycling practices. A total of 359 usable responses were collected through the survey method, which exceeds the minimum requirement for structural equation modeling (SEM). According to Hair et al. (2010), a minimum of 200 respondents is generally sufficient for SEM; however, this recommendation is a guideline rather than a fixed rule. For greater statistical power, the adequacy of the sample size was further supported using G*Power analysis for SEM models, which indicated that a minimum sample of approximately 280 participants is required to detect medium effect sizes with 0.80 power at the 5% significance level. Therefore, the final sample size of 359 not only meets but exceeds the statistical requirement, enhancing the reliability and generalizability of the findings. In addition to statistical sufficiency, the sample is diverse in terms of age, gender, experience, education levels, and company sizes, which improves the representativeness of the sample and reduces potential sampling bias. The purposive sampling method used across different regions and company sizes further strengthens the validity of the results by ensuring inclusion of various sub-sectors within the construction industry.

### Data collection and data analysis method

In this research, data were collected from workers in the Chinese construction industry with a structured questionnaire as the primary survey instrument. A translation–back translation process (Brislin, 1980) was employed to enhance the contextual and linguistic validity of the Chinese version for the construction industry. After translation, the questionnaire was reviewed by academic experts and industry professionals to ensure clarity, cultural appropriateness, and face validity. Subsequently,

The data collection was done in two stages: a pilot study and the final survey. Grimm (2010) indicates that pilot testing was conducted to measure the content validity of the instrument and to recognize typical problems like item misinterpretation, reluctance to respond, or too much completion time. The pilot test respondents, as recommended by Malhotra et al. (2004), were taken from the same population, i.e., construction workers in China. This phase selected a purposive sample of 30 workers for the research. The results showed that the items in the questionnaire were very easy and they took about 15 min to complete on average, with item comprehension and initial reliability, with Cronbach’s alpha values for all constructs exceeding 0.70. These steps confirm that the instrument is psychometrically equivalent to its original version and suitable for the local context.

In the final data collection phase, self-administered surveys were used to maximize accessibility and participation. To reduce the risk of bias, which are the inherent challenges in survey-based research, several design strategies were put into place. Sampling bias was dealt with by recruiting respondents from different works, roles, organizations, and areas of the industry, thus ensuring a fair representation of demographics, project sizes, and organization types. Non-response bias was neutralized by producing a short questionnaire (12–15 min). To ensure the validity of the questionnaire, the scale was designed by adapting items from existing, well-established, and validated scales (Ioannou et al., 2013; Wu et al., 2017; Superti et al., 2021), providing assurance of strong theoretical and empirical bases so as to strengthen the overall psychometric properties of the measures.

The collected data were analyzed using the Statistical Package for the Social Sciences (SPSS) to examine demographic characteristics. The latest version of AMOS was also utilized for the advanced statistical testing. Confirmatory Factor Analysis (CFA) were applied to check the underlying factor structure and to confirm the measurement model.

### Measurement scales

The scales used to measure each of the Theory of Planned Behavior (TPB) constructs were further detailed as follows: Recycling Behavior (RB) was measured with seven items, which were taken from another study (Wu et al., 2017). Attitude (AT), Subjective Norms (SN), and Perceived Behavior Control (PBC) used 6, 4, and 5 items, which were assigned, respectively, following the scale of Ioannou et al. (2013). For response on the Intention to Recycle (IR), Consequences/ Outcome of Recycling (COR), and Past Recycling Behavior (PRB), 3, 5, and 6 items were then used, respectively. These are also adopted from the scale of Ioannou et al. (2013). To collect responses on the Consequences to Sustainability (CS), 2 items were used from the scale following that of Superti et al. (2021).

### Questionnaire design

The questionnaire comprised two sections. The first section consisted of the demographic profile of the respondents, while the second section contained the constructs of the TPB, including statements to measure the sample responses on variables. To account for bias in the survey results that could occur due to the sequence of questions, behavior-related questions were placed before attitude-related questions. Responses were recorded on a 5-point Likert-type rating scale with the statements anchored as strongly agree to strongly disagree.

It is important to address common method bias during questionnaire design, as recommended by academic scholars. Common method bias refers to the incorrect variance that occurs due to the measurement method rather than the measurement of the constructs (Podsakoff et al., 2003). When the same method is used for measuring dependent and independent variables, it can cause measurement errors that negatively impact the validity of the results (Chang et al., 2010). Various approaches can minimize or reduce common method bias. In this study, during questionnaire design, the issue was addressed by using different sources of information in constructing the variables. Moreover, social desirability refers to respondent-related sources of bias (error). In behavioral research studies, social desirability bias occurs when respondents answer in a socially desirable manner (Lavrakas, 2008). Some respondents do this to create a positive image of themselves, which can lead to over-reporting of results. To avoid this bias, the questionnaire was designed to ensure the confidentiality and anonymity of respondents and used an indirect (passive) questioning format (Grimm, 2010).

## Results

The data collected in this study were analyzed using both SPSS and AMOS to test the measurement and structural models. Initially, descriptive statistics were used to summarize the demographic characteristics of the respondents. Following this, Confirmatory Factor Analysis (CFA) was conducted to assess the validity of the measurement model. CFA confirmed that the constructs met the required thresholds for convergent and discriminant validity based on factor loadings, composite reliability (CR), and average variance extracted (AVE). To test the hypothesized relationships among the constructs, Structural Equation Modeling (SEM) was applied. Model fit indices, including the chi-square/degree of freedom (CMIN/df), Comparative Fit Index (CFI), Incremental Fit Index (IFI), and Root Mean Square Error of Approximation (RMSEA), were used to confirm the adequacy of the structural model. The model demonstrated an excellent fit, with all indices falling within acceptable ranges.

Table 1 shows the results to understand the distribution of sample with reference to the demographics variables descriptive analysis was conducted using SPSS. Clearly, regarding gender, males comprised 62% of the study sample, while the remaining 37.6% were females. Almost half of the respondents were above 40 years of age, followed by the age group of 36–40 years. Only 12 and 13% of the employees fell in the age groups of 18–25 and 26–35 years, respectively. In terms of working experience, 50% of the employees had worked for more than 12 years, while just 12% of the respondents had work experience of less than 3 years. Regarding education, 39% employees possessed Master Degree while 45% had Bachelor Degree. Only 16% of the sample had attended up to High School. Company size was categorized into four groups, namely 1–50, 51–100, 101–200 workers, and more than 200 workers. It is found that majority of the sample worked in large-sized firms of more than 200 employees. On the other hand, 18% of the respondents worked in small-sized firms with less than 50 workers. Table 2 shows the factor loadings that measures Reliability, Convergent, and Discriminant Validity.

**TABLE 1:** Demographic profile of the respondents.

Indicators	Dimensions	Frequency	%
Gender	Male	224	62.4
	Female	135	37.6
	Total	359	100.0
Respondents’ age	18–25	43	12.0
	26–35	48	13.4
	36–40	86	24.0
	Above 40	182	50.7
	Total	359	100.0
Experience	Below 3 years	45	12.5
	4–7 years	71	19.8
	8–12 years	63	17.5
	Above 12 years	180	50.1
	Total	359	100.0
Education	High school or below	57	15.9
	Bachelor	162	45.1
	Master	140	39.0
	Total	359	100.0
Company size	1–50 employees	65	18.1
	51–100 employees	92	25.6
	101–200 employees	95	26.5
	More than 200 employees	107	29.8
	Total	359	100.0

**TABLE 2:** Reliability and convergent validity.

Variable	Items	Loading	CR	AVE	MSV
Recycling behavior (RB)	RB1	0.819	0.922	0.628	0.298
	RB2	0.732			
	RB3	0.774			
	RB4	0.810			
	RB5	0.837			
	RB6	0.783			
	RB7	0.758			
Attitude (AT)	AT1	0.827	0.910	0.627	0.135
	AT2	0.794			
	AT3	0.809			
	AT4	0.815			
	AT5	0.767			
	AT6	0.735			
Consequences/ outcome of recycling (COR)	COR1	0.902	0.827	0.503	0.108
	COR2	0.909			
	COR3	0.597			
	COR4	0.454			
	COR5	0.470			
Past recycling behavior (PRB)	PRB1	0.604	0.868	0.632	0.101
	PRB2	0.580			
	PRB3	0.948			
	PRB4	0.933			
Subjective norms (SN)	SN1	0.837	0.849	0.593	0.070
	SN2	0.964			
	SN3	0.558			
	SN4	0.593			
Perceived behavioral control (PBC	PBC1	0.406	0.835	0.530	0.096
	PBC2	0.457			
	PBC3	0.629			
	PBC4	0.958			
	PBC5	0.970			
Intention to recycle (IR)	IR1	0.844	0.864	0.680	0.135
	IR2	0.847			
	IR3	0.780			
Consequences to sustainability (CS)	CS1	0.896	0.904	0.824	0.298
	CS2	0.920			

As the items were selected from existing tools and a new tool was constructed, confirmatory factor analysis was executed to establish its validity using AMOS. In order to measure the Convergent Validity, three conditions need to be satisfied. Firstly, the factor loadings must be high, and secondly, the Composite Reliability values must score above 0.7. Lastly, the AVE values must exceed 0.5. Table 2 shows that all the study items loaded significantly against the constructs as their loadings are more than 0.5. Moreover, the Composite Reliability (CR) values are also above 0.7. The values for AVE are found to be larger than 0.5, hence the Convergent Validity is proven and fulfills the criteria of Fornell and Larcker (1981) and Hair et al. (2019a).

Table 2 presents the results of the measurement model assessment, focusing on the reliability and convergent validity of the constructs used in the study. The factor loadings for most of the items are above the acceptable minimum threshold of 0.50, indicating that each item significantly contributes to its respective construct, ensuring high validity of the data set. The recycling behavior (RB) items demonstrate strong loadings ranging from 0.732 to 0.837, confirming that the items adequately capture the underlying recycling behavior construct. However, the items COR 4 and 5 did not load within the acceptable range; therefore, they were not further added to the composite score (Hair et al., 2019b). Similarly, the attitude (AT) items also show robust loadings between 0.735 and 0.827, reinforcing their measurement accuracy. Although some items in the consequences/outcome of recycling (COR) and perceived behavioral control (PBC) constructs have comparatively lower loadings, they still meet the minimum requirement for inclusion. The composite reliability (CR) values for all constructs exceed the recommended threshold of 0.70, further confirming internal consistency. For example, RB shows a CR of 0.922 and AT shows a CR of 0.910, both indicating excellent reliability. This demonstrates that the items within each construct consistently measure the same underlying concept.

Furthermore, the Average Variance Extracted (AVE) values across all constructs exceed the acceptable limit of 0.50, except for some borderline cases in COR and PBC, where lower-loading items slightly reduce AVE values but still remain acceptable. An AVE above 0.50 suggests that each construct explains more than half of the variance in its items, establishing convergent validity. The Maximum Shared Variance (MSV) values for each construct are lower than their corresponding AVE values, confirming discriminant validity according to Fornell and Larcker’s criteria. This indicates that each construct is distinct from the others and measures a unique aspect of the theoretical model. Overall, the results in Table 2 confirm that the measurement scales used in the study are both reliable and valid, providing a solid foundation for further structural equation modeling and hypothesis testing. Table 3 shows the values for self and inter-item correlations of the constructs. According to the results, the self-correlations were high between the constructs, while the inter-construct correlations were lesser than their self-correlations, confirming the Discriminant Validity.

**TABLE 3:** Discriminant validity.

Variables	RB	AT	COR	PRB	SN	PBC	IR	CS
RB	0.792							
AT	0.080	0.792						
COR	0.176**	0.224***	0.709					
PRB	−0.024	0.318***	0.177**	0.795				
SN	0.160**	0.265***	0.127*	0.110	0.770			
PBC	0.082	0.230***	0.164**	0.167**	0.201**	0.728		
IR	0.151*	0.367***	0.329***	0.159**	0.232***	0.310***	0.825	
CS	0.546***	−0.060	0.053	−0.136*	0.057	−0.034	−0.069	0.908

*p < 0.05;

**p < 0.01;

***p < 0.001.

To establish the validity of every subscale, discriminant validity is calculated using SEM AMOS to measure the extent to which each individual subscale is different from the other and to measure the relevant construct that it is intended to measure. Table 3 presents the discriminant validity assessment by comparing the square roots of the Average Variance Extracted (AVE) for each construct (diagonal values) with the inter-construct correlations (off-diagonal values). Discriminant validity is established when the diagonal values are higher than the correlations between constructs, indicating that each construct is empirically distinct and captures unique aspects of the theoretical framework. For example, the square root of AVE for Recycling Behavior (RB) is 0.792, which is greater than its correlations with other constructs, such as Attitude and Intention to Recycle. Similarly, the square root of AVE for Intention to Recycle (IR) is 0.825, which is also higher than its correlations with other constructs like Perceived Behavioral Control (0.310) and Consequences/Outcome of Recycling (0.329). These results confirm that the constructs are not only related but also sufficiently independent from one another, supporting the discriminant validity of the measurement model. This ensures that the observed relationships in the structural model are not a result of overlapping measurements but rather reflect true conceptual distinctions among the variables studied

Table 4 below shows the values of the indices used for finding the extent of the model fitness. The indices used were CMIN/df, CFI, IFI and RMSEA. The table also shows the threshold values for accurate interpretation.

**TABLE 4:** Model fit measures.

Measure	Observed estimate	After modification	Threshold	Interpretation
CMIN	1,879.109	915.279	–	–
DF	566.000	561	–	–
CMIN/DF	3.320	1.632	Between 1 and 3	Excellent
CFI	0.847	0.959	>0.95	Excellent
IFI	0.848	0.959	>0.95	Excellent
RMSEA	0.081	0.042	<0.06	Excellent

Table 4 presents the model fit measures, which assess how well the proposed structural model aligns with the observed data using AMOS. Initially, the model showed suboptimal fit, with a CMIN/DF (chi-square divided by degrees of freedom) value of 3.320, slightly exceeding the preferred range of 1–3, and CFI and IFI values of 0.847 and 0.848, respectively, falling below the acceptable threshold of 0.95. The RMSEA value was also relatively high at 0.081, indicating a mediocre fit. However, after model modifications, the fit indices improved significantly. The adjusted CMIN/DF dropped to 1.632, well within the excellent range, while both the CFI and IFI increased to 0.959, surpassing the minimum recommended value of 0.95, indicating a strong model fit. Additionally, the RMSEA decreased to 0.042, comfortably below the 0.06 threshold, further confirming the model’s excellent fit. Overall, the post-modification results demonstrate that the refined model provides a highly accurate representation of the data structure, supporting the model’s validity for further hypothesis testing.

**FIGURE 3:**
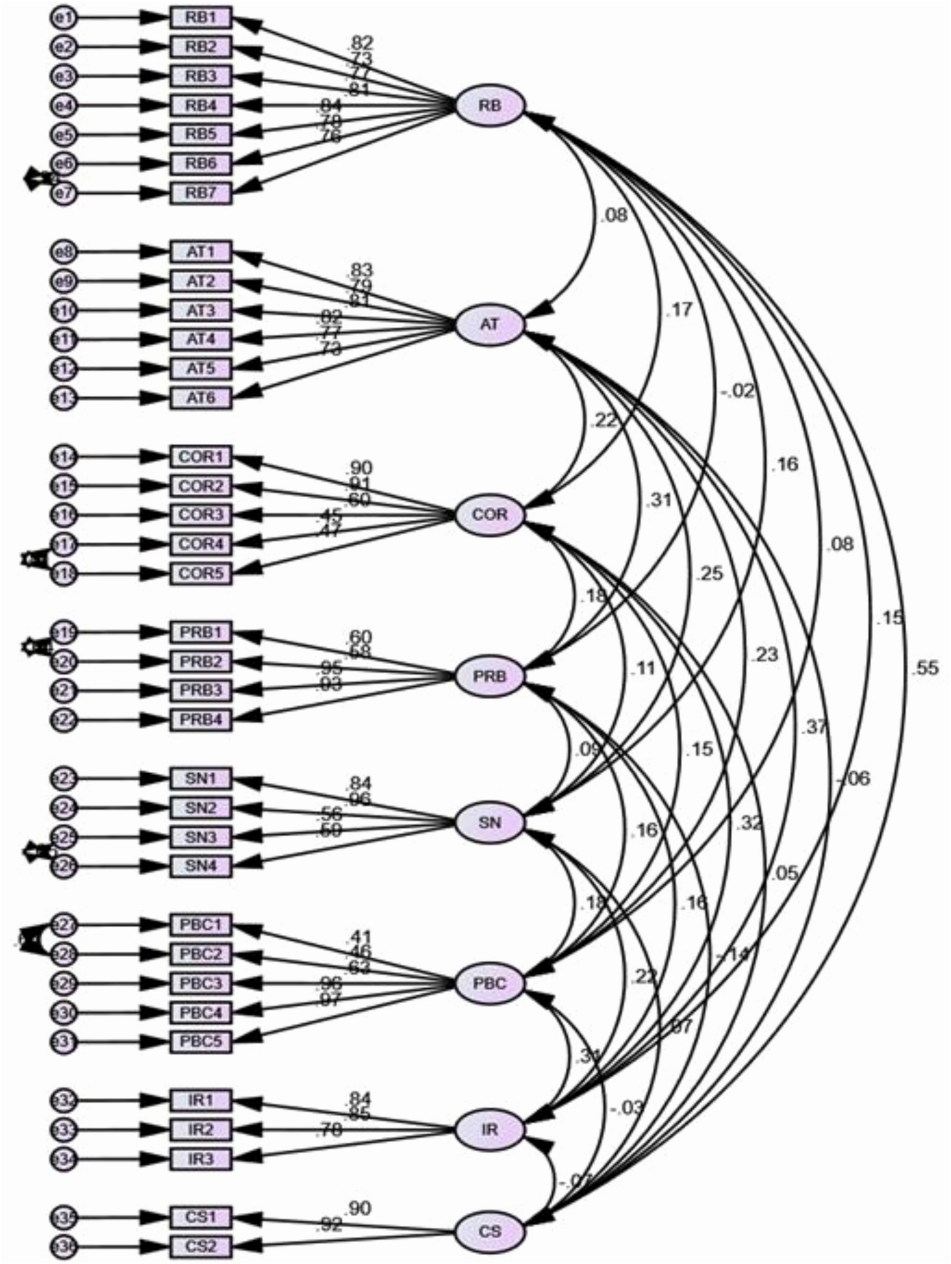
Measurement model.

Figure 3 is the measurement model that illustrates the model clearly.

Figure 3 illustrates the measurement model derived from Confirmatory Factor Analysis (CFA), which validates the reliability and validity of the constructs used in the study. Each latent variable (e.g., Attitude, Subjective Norms, Perceived Behavioral Control, etc.) is represented by its respective observed indicators, and the factor loadings between them show the strength of association. The figure confirms that the observed variables effectively represent their corresponding constructs, supporting convergent validity. The model demonstrates a good overall fit, as evidenced by the refined fit indices (e.g., CFI = 0.959, RMSEA = 0.042), which fall within acceptable thresholds, thereby establishing the credibility of the measurement structure for the subsequent structural analysis.

Table 5 shows the paths of the deduced hypotheses of this study. To evaluate the predicting role of the core factor in intentions to recycle using structure equation modeling through AMOS. Beta estimates and *p*-values were employed to either accept or reject the seven hypotheses. The impact of age, gender and experience as the control variables were tested to evaluate their respective significance related to each hypothesis.

**TABLE 5:** Hypothesis testing.

Hypothesis	Hypothetical path			Estimate	S.E.	C.R.	*P*	Hypothesis support
H1	AT	→	IR	0.184	0.046	3.976	0.000	Yes
H2	SN	→	IR	0.077	0.046	1.662	0.096	No
H3	PBC	→	IR	0.272	0.054	5.046	0.000	Yes
H4	COR	→	IR	0.215	0.056	3.859	0.000	Yes
H5	PRB	→	IR	−0.013	0.040	−0.317	0.752	No
H6	IR	→	RB	0.132	0.051	2.608	0.009	Yes
H7	RB	→	CS	0.777	0.071	10.974	0.000	Yes
**H8-control variables (socio-demographic attributes)**								
	Age	→	RB	0.059	0.029	1.999	0.046	Significant
	Gender	→	RB	−0.020	0.064	−315	0.753	Not Significant
	Experience	→	RB	0.061	0.028	2.166	0.030	Significant

Table 5 presents that H1, which predicts the path of AT with IR, is accepted. A positive attitude toward recycling significantly increases the intention to recycle. This suggests that individuals who view recycling favorably are more likely to plan to engage in recycling behaviors. The second hypothesis (H2) is rejected. Subjective norms do not significantly influence the intention to recycle. This implies that subjective norms may not strongly motivate individuals to plan to recycle in this study. H3 shows a direct relationship between PBC and IR, indicating that Greater perceived behavioral control, such as feeling capable of recycling due to access or knowledge, significantly increases the intention to recycle. This suggests that individuals who believe recycling is easier or within their control are more likely to intend to recycle. H4 is accepted. Concern for the outcomes of recycling, such as environmental or social benefits, significantly increases the intention to recycle. Individuals who care about the positive impacts of recycling are more likely to plan to recycle. However, the fifth hypothesis (H5) is rejected. Perceived risks or barriers, such as inconvenience or effort, do not significantly affect the intention to recycle. This suggests that barriers may not strongly deter individuals from planning to recycle in this context. The last two hypotheses, i.e., H6 and H7, are accepted. A stronger intention to recycle significantly increases actual recycling behaviors. Individuals who plan to recycle are more likely to follow through with recycling actions. Similarly, engaging in recycling behaviors significantly leads to positive outcomes, such as personal satisfaction or environmental benefits. This highlights that recycling actions result in meaningful consequences

Results also indicated that age was a significant predictor of recycling behavior than older workers had more recycling behaviors than younger. Similarly, workers with more experience have more recycling behaviors, while gender was not a significant predictor of recycling behaviors.

**FIGURE 4:**
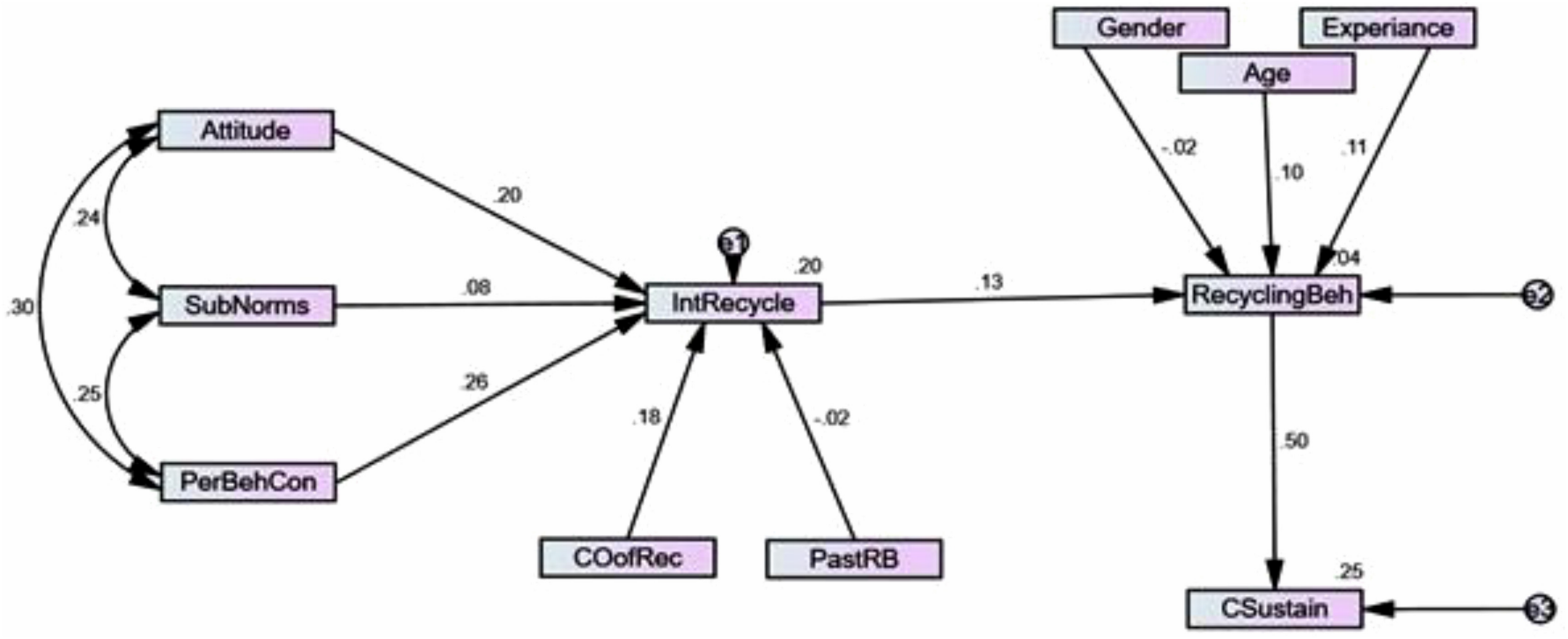
The structural model.

Figure 4 presents the structural model showing the tested hypothesized relationships among the extended Theory of Planned Behavior (TPB) constructs. It visually depicts the significant and non-significant paths with directionality, path estimates, and hypothesis labels (H1 to H7). The model confirms that Attitude, Perceived Behavioral Control, and Consequences of Recycling significantly predict Intention to Recycle (IR), while Subjective Norms and Past Recycling Behavior do not. Moreover, IR positively influences Recycling Behavior (RB), which in turn has a strong, significant impact on Consequences to Sustainability (CS). The model also highlights the moderating influence of socio-demographic factors such as age and experience on recycling behavior (H8). This structural path diagram reinforces the extended TPB’s explanatory power in predicting recycling behavior within the Chinese construction industry.

## Discussion

To identify the factors influencing recycling behavior in the Chinese construction sector, this research extended the Theory of Planned Behavior (TPB) by including two new constructs: Consequences/Outcomes of Recycling (COR) and Consequences to Sustainability (CS). It has also been proven in previous research that the predictive power of TPB can be improved by incorporating such variables. For example, Tommasetti et al. (2018) used Consequences to Sustainability as part of their Extended TPB in a study of customers’ attitudes toward restaurant sustainability, and Mohamad et al. (2022) used Consequences/Outcomes of Recycling and demographic factors in a study of consumers’ recycling of e-waste. This follows Ajzen’s (1991) contention that augmenting TPB with more predictors enhances the theory’s explanatory power.

The results of the present study identify several key contributions. Attitude was first revealed as a strong predictor of recycling intention, as was seen in previous studies (Ma and Zhang, 2020; Wan et al., 2021; Greaves et al., 2013), which established environmental attitudes to be the main predictor of sustainable behavior. Construction workers are more likely to plan to recycle if they find it to be ethically correct and good for the environment. Second, perceived behavioral control was supported as a valid role, consistent with Ajzen (1991), Tonglet et al. (2004b), Mak et al. (2019), and Liu et al. (2020), indicating that recycling behavior is greatly impacted by perceptions of time, resources, and organizational support.

Third, empirically, the present study supported the predictive function of consequences/outcomes of recycling, where those who saw more economic, environmental, and organizational benefits were more likely to recycle. This result agrees with Zhang et al. (2021) and Lindsay and Strathman (1997), who stressed that behavioral decisions tend to be directed by perceived long-term consequences.

However, past recycling behavior and subjective norms did not predict the intention to recycle significantly. This is in contrast to research in household and consumer contexts (Liu et al., 2020; Mohamad et al., 2022; Geiger et al., 2019), in which social habits and influence were found to be significant predictors. One potential reason is the Chinese construction industry’s highly structured, hierarchical, and results-oriented nature. Staff can prioritize work and project achievement over social adjustment, while high staff turnover and changing requirements of the projects may be challenging continuity in recycling behavior.

The extended TPB model was also confirmed through the relationship between recycling intentions, behavioral recycling, and their wider sustainability consequences. This is in support of the argument that recycling activities are not merely short-term behavioral responses but also feed into systemic sustainability objectives. These conclusions are corroborated with Kumar (2019) and Savari and Khaleghi (2023), whose research showed the role of pro-environmental behaviors toward long-term sustainability.

Even though recycling is being recognized as a sustainable activity, major challenges persist. In China, for instance, fewer than 5% of construction waste is recycled, while in developed nations 70–95% is recycled (Hua et al., 2022; Jin et al., 2017). This is one of the most important implementation gaps in waste management practices. This gap is filled by the present research through investigating the psychological and behavioral determinants of recycling in the construction sector, thus contributing to theory and practice in sustainable waste management.

## Conclusion

This study is an attempt to examine Recycling Behavior (RB) in the construction sector by extending the basic TPB framework. The variable RB is considered the dependent variable of the study, and data were collected to see its determinants. The study resolves that Attitudes and Behavioral Control are concerned with the intention and actual behaviors exhibited by the construction workers. This research advances both theory and practice. Theoretically, it enhances TPB by demonstrating that recycling practices have sustainability implications in addition to being dependent on internal factors. Traditional postulates are violated by the absence of relevance of SN and PRB, which suggests that TPB may operate differently in collectivist but highly task-oriented industries. From a practical standpoint, the consequences provide managers and policymakers with guidance. Emphasis should be placed on encouraging a positive outlook through training programs, awareness campaigns, and recycling activities.

Increasing employees’ perception of control by providing sufficient facilities, resources, and organizational support is also crucial. With this research future interventions must be adjusted for age and experience levels, since older and more experienced workers showed more robust recycling behaviors. The results support the extended TPB, whereby both the internal and external forces have the propensity to affect an individual’s choice to carry out a certain adoption of behavior. Even though Subjective Norms (SN), frequency of prior behavior, and gender had no significant role, the other factors determined the outcomes of the Recycling Behavior (RB). As a result of the individual’s actions, both internal and external behavioral drivers experienced a feedback loop, influencing the individual’s future decisions to behave in a certain manner.

The factors influencing the adoption of recycling behavior in using recycled construction materials in their specific contexts have been found to make a significant contribution. In terms of the transformation and disposal of construction waste, China can be considered representative of the rest of the world. As a result, an empirical analysis of the Chinese example could help shed light on the global shift in attitudes toward construction waste and may assist in managing recycling policies for different countries. In conclusion, analyzing the patterns of China’s construction waste over time and space, establishing a connection between the growth of the construction industry and its evolving traits, and laying a foundation for recycling decisions could be highly valuable both theoretically and practically for the rest of the world. Therefore, the findings of this study could serve as a basis for recommendations that other construction firms in different countries might apply. China’s case could also be highlighted to encourage workers’ awareness of the importance of recycling behavior, especially in reducing waste by recycling behavior. At the same time, these efforts are crucial for protecting the environment and conserving natural resources. Ultimately, they will promote higher sustainability and help achieve the UN’s Sustainable Development Goals. Overall, this research contributes to the extended TPB model by setting out the role of attitudes, perceived control, and outcome awareness in determining recycling behaviors and by connecting recycling behaviors with sustainability.

### Research contributions

This research contributes at three levels: theoretical, practical, and policy, which greatly increases its overall impact. The study theoretically offers a substantial empirical validation of an Extended Theory of Planned Behavior (TPB) framework within the inadequately explored domain of the Chinese construction industry. By effectively integrating and evaluating two innovative constructs—Consequences/Outcomes of Recycling (COR) and Consequences to Sustainability (CS)—the model’s explanatory capacity is augmented, transcending intention-formation to encompass the feedback loop connecting behavior to overarching sustainability objectives. Moreover, the results contest traditional Theory of Planned Behavior (TPB) assumptions by demonstrating that Subjective Norms (SN) and Past Recycling Behavior (PRB) are not substantial predictors within this particular industrial and cultural context. This nuanced insight represents a significant theoretical progression, indicating that the relevance of TPB constructs is contingent upon context, especially in industries characterized by a strong focus on tasks and hierarchical structures, where peer pressure and habitual behaviors may be eclipsed by pragmatic attitudes, perceived control, and outcome expectations. The study provides construction managers and companies with practical tools to enhance their waste management performance. The results clearly show that interventions should focus on building positive attitudes through targeted training and awareness campaigns, while also improving Perceived Behavioral Control (PBC) by making sure that recycling infrastructure is available, giving clear instructions, and getting rid of operational barriers. The substantial impact of COR indicates that conveying the concrete environmental and economic advantages of recycling can effectively incentivize employees. The findings offer an evidence-based justification for governmental and industrial entities to transcend generic mandates at the policy level. Policies can be improved to give companies that invest in the key drivers—education programs that change attitudes and infrastructure that gives people more control—more reasons to do so. Furthermore, acknowledging the considerable impact of age and experience, policymakers and organizations can formulate customized communication and training strategies, concentrating engagement initiatives on younger and less experienced groups to cultivate a lasting recycling culture, thereby advancing national waste reduction and sustainability objectives.

### Limitations

This study highlights possible avenues for future research via underscoring its limitations. The first limitation is that it is confined only to the construction sector. Future studies could venture into other sectors such as manufacturing, IT, and textiles. Secondly, as the study is only in China, it is recommended that data be obtained from other nations to conduct a cross-country comparison and to analyze which nation has workers with the most Recycling Behaviors. This turn will exhibit outcomes that relate to the significance of the Recycling Behaviors. Thirdly, the present research employed cross-sectional data. However, a longitudinal study may be carried out to see the varying after-effects of Recycling Behaviors. Fourthly, employing a more robust statistical method, such as an experimental study, would be more interesting to figure out other influential factors to gain a deeper understanding of the behaviors. Finally, the TPB framework must be extended further to investigate the role of other behavioral and demographic factors to see their impacts.

## Data Availability

The original contributions presented in the study are included in the article; further inquiries can be directed to the corresponding author.
